# The influence of adult hip shape genetic variants on adolescent hip shape: Findings from a population-based DXA study

**DOI:** 10.1016/j.bone.2020.115792

**Published:** 2021-02

**Authors:** Monika Frysz, Denis Baird, Jenny S. Gregory, Richard M. Aspden, Nancy E. Lane, Claes Ohlsson, Ulrika Pettersson-Kymmer, David Karasik, Jonathan H. Tobias, Lavinia Paternoster

**Affiliations:** a Musculoskeletal Research Unit, Translational Health Sciences, Bristol Medical School, University of Bristol, Bristol, UK; b Medical Research Council Integrative Epidemiology Unit, Population Health Sciences, Bristol Medical School, University of Bristol, Bristol, UK; c Centre for Arthritis and Musculoskeletal Health, University of Aberdeen, Aberdeen, UK; d Center for Musculoskeletal Health, University of California Davis School of Medicine, Sacramento, CA, USA; e Centre of Bone and Arthritis Research, Department of Internal Medicine and Clinical Nutrition, Institute of Medicine, Sahlgrenska Academy, University of Gothenburg, Gothenburg, Sweden; f Department of Drug Treatment, Sahlgrenska University Hospital, Region Västra Götaland, Gothenburg, Sweden; g Clinical Pharmacology, Department of Integrative Medical Biology, Umeå University, Umeå, Sweden; h Hinda and Arthur Marcus Institute for Aging Research, Hebrew SeniorLife, Boston, MA, USA; i The Azrieli Faculty of Medicine, Bar-Ilan University, Safed, Israel

**Keywords:** ALSPAC, DXA, Hip shape, Osteoarthritis, Hip fracture risk, Genetic association

## Abstract

**Objective:**

Hip shape is a well-recognized risk factor for hip osteoarthritis (OA) and hip fracture. We aimed to investigate whether the genetic variants known to be associated with adult hip shape were also associated with adolescent hip shape.

**Methods:**

Hip DXA scans, obtained in offspring from the Avon Longitudinal Study of Parents and Children (ALSPAC) at two time points (mean ages 13.8 and 17.8 years), were used to quantify hip morphology using a 53-point Statistical Shape Model (SSM). Principal component analysis was used to generate hip shape modes (HSMs). Genetic variants which had previously shown genome-wide significant association with specific HSMs in adults were tested for association with the same HSMs in adolescents (at each timepoint separately) using SNPTEST v2.

**Results:**

Complete genotypic and phenotypic data were available for 3550 and 3175 individuals at 14 and 18 years, respectively. The strongest evidence for association with adolescent hip shape was for a variant located near *SOX9* (rs2158915) with consistent effects across both time points for HSM1 (age 14: beta −0.05, *p* = 9.9 × 10^−8^; age 18: −0.05, *p* = 3.3 × 10^−6^) and HSM5 (age 14: beta −0.07, *p* = 1.6 × 10^−4^; age 18: −0.1, *p* = 2.7 × 10^−6^). There was also strong evidence of association between rs10743612 (near *PTHLH*) and HSM1 (age 14: 0.05, *p* = 1.1 × 10^−5^; age 18: 0.04, *p* = 0.003) and between rs6537291 (near *HHIP*) and HSM2 (age 14: −0.06, *p* = 0.001; age 18: −0.07, *p* = 0.001) across both time points. The genes with the strongest associations with hip shape in adolescents, (*SOX9*, *PTHLH* and *HHIP*) are known to be involved in endochondral bone formation. HSM1 indicates narrower aspect ratio of the upper femur, whereas both HSM2 and HSM5 reflect variation in the femoral head size and femoral neck width, features previously found to be related to the risk of OA in later life. The *SOX9* locus has previously been found to associate with increased risk of hip fracture.

**Conclusion:**

In conclusion, variants implicated in endochondral bone formation appear to consistently influence hip shape between adolescence and adulthood, including those aspects related to risk of hip OA and/or fracture in later life.

## Highlights

• Genetic variants for adult hip shape are associated with DXA-derived hip shape in adolescents.
• Genetic influences on hip shape are present as early as 14 years of age.
• Variants implicated in endochondral bone formation may play a role in hip shape across lifespan.

## 1 Introduction

Hip shape is thought to be one of the most important risk factors for hip osteoarthritis (OA) [1], and has also been found to be associated with the risk of osteoporotic hip fracture [2]. Whilst severe skeletal disorders such as developmental dysplasia of the hip (DDH) are known to be associated with early-onset OA [3], a growing body of evidence suggests that more subtle changes in joint morphology play an important role in the development of hip OA [1]. Whereas hip geometry (including measures such as femoral neck width (FNW) or neck shaft angle [4,5]) are well known to be related to hip fracture, a relationship between proximal femur shape, measured with statistical shape model (SSM), and hip fracture has also been reported [6].

SSM was first introduced by Gregory et al. [2], as a means of capturing the global shape of the proximal femur, to investigate the relationship with osteoporotic hip fracture. Since then this method has been applied to radiographs to investigate associations with incident hip fracture [6], incident radiographic hip OA (RHOA) [7] and to predict total hip replacement (THR) in OA cases [8,9]. More recently, this method has been successfully applied to dual-energy X-ray absorptiometry (DXA) hip scans to study genetic influences on hip shape in adults, in the first genome-wide meta-analysis of hip shape [10]. Other studies of DXA-derived hip shape reported associations between previously established OA loci and hip shape [11] as well as associations between hip shape and radiographic and symptomatic hip OA [12]. Further genetic evidence for the involvement of joint shape in the development of OA comes from a recent genome-wide association study (GWAS) of 770 cases with DDH and 3364 healthy controls, in the National Joint Registry for England, Wales, Northern Ireland and the Isle of Man (NJR), which identified rs143383 variant in *GDF5 to be robustly associated with DDH case status* [13]; the same variant has also been reported to be associated with increased risk of OA [14]. These findings are consistent with the suggestion that OA development is mediated through joint shape and variants associated with hip morphology could contribute to the development of OA.

With studies investigating genetic influences on hip shape in older adults, it is difficult to distinguish if genetic associations exist with hip shape before the onset of disease, or with changes occurring as a result of OA. In order to bridge this knowledge gap, we investigated if the eight genetic variants associated with adult DXA-derived hip shape in the previous adult GWAS [10] are also associated with hip shape in adolescence. To enable genetic associations to be directly compared, the same SSM template was used to evaluate hip shape in adults and adolescents.

## 2 Methods

### 2.1 Study participants – the Avon Longitudinal Study of Parents and Children (ALSPAC)

Pregnant women resident in Avon, UK with expected dates of delivery 1st April 1991 to 31st December 1992 were invited to take part in the study. The initial number of pregnancies enrolled was 14,541 (for these at least one questionnaire has been returned or a “Children in Focus” clinic had been attended by 19/07/99). Of these initial pregnancies, there was a total of 14,676 foetuses, resulting in 14,062 live births and 13,988 children who were alive at 1 year of age. In addition to the initial enrolment, further recruitment took place when the children were on average 7 years old, and another from age 8 onwards to which eligible children and those not initially enrolled were also invited. This resulted in a total of 15,247 pregnancies enrolled [15,16]. The study website contains details of all the data that is available through a fully searchable data dictionary and variable search tool (http://www.bristol.ac.uk/alspac/researchers/our-data/). The present study uses data from Teen Focus (TF) 2 and TF 4 follow-up research clinics, undertaken when the offspring were on average 13.8 and 17.8 years old. Of 11,351 individuals invited to the TF 2 clinic, 6147 attended and of 10,101 individuals invited to the TF 4 clinic, 5217 attended. Given that some participants attended TF 2 and not TF 4 clinic, and vice versa, each time point was analysed separately. Ethical approval for the study was obtained from the ALSPAC Ethics and Law Committee and the Local Research Ethics Committees.

### 2.2 Statistical shape model

Hip DXA scans of the ALSPAC offspring, performed using GE Lunar Prodigy (Madison, WI, USA), were used to quantify the shape of proximal femur. All images were uploaded into Shape software (University of Aberdeen) and marked up with a set of landmark points outlining the proximal femur and acetabular sourcil (eyebrow). Details regarding the method and derivation of data in ALSPAC offspring have been described in more detail previously [17]. Briefly, following the placement of 53 landmark points, Procrustes and principal component analyses (PCAs) were used to generate 10 linearly independent modes of variation (hip shape modes (HSMs)). In order to allow direct comparison of results with adult population, the same SSM template which was used to investigate associations between genetic variants and adult hip shape in a recent genome-wide meta-analysis [10] (results of which are used in this study) was applied to adolescent data. Following Procrustes and PCA at each adolescent time point, adult model (based on DXA images from adult cohorts) was applied to adolescent data and HSMs were re-calculated. HSMs are therefore expressed as deviation from the mean adult shape. These variables had mean of 0 and SD of 1 in the adult sample, but their distributions differ when applied to the adolescent data (see Table 1 for details). Nevertheless, when assessing potential loss of independence of the modes, following application of the adult model, a minimal loss of independence was seen, as described previously [17]. In individuals with outcome data available at both timepoints (see Supplementary Table 1), correlation coefficients for HSMs at age 14 vs age 18 years ranged from 0.30 to 0.79, indicating a moderate to high degree of correlation as expected.

### 2.3 Genome-wide genetic data

A total of 9912 ALSPAC offspring were genotyped using the Illumina HumanHap550 quad genome-wide SNP genotyping platform (Illumina Inc., San Diego, CA, USA) at Laboratory Corporation of America (LabCorp Holdings, Burlington, NC, USA) by 23andMe. PLINK software (v1.07) was used to carry out quality control [18]. Individuals were excluded on the basis of gender mismatches, minimal or excessive heterozygosity, disproportionate levels of individual missingness (>3%), evidence of cryptic relatedness (>10% of alleles identical by descent (IBD)) and being of non-European ancestry (assessed by multidimensional scaling analysis seeded with HapMap2 individuals). SNPs with a minor allele frequency of <1% and call rate of <95% were removed. Furthermore, only SNPs that passed an exact test of Hardy–Weinberg equilibrium (*p* > 5 × 10^−7^) were considered for analysis. SNPs with genotype missingness >1% were removed. Genetic imputation was performed using Impute2 v2.2.2 software using the 1000 genomes reference panel (phase 1, version 3) using 2186 haplotypes from all populations (haplotype release date Dec 2013), which resulted in 28,699,419 SNPs available for analysis. A total of 8237 unrelated individuals with available genotype data were available for analysis.

**Table 1:** Descriptive statistics of study participants.

“Age 14”	Both	Females	Males
	N = 3550	N = 1847	N = 1703
	Mean (SD)	Mean (SD)	Mean (SD)
Age (years)	13.8 (0.20)	13.8 (0.20)	13.8 (0.20)
Height (cm)	163.5 (7.6)	162.1 (6.2)	165.0 (8.7)
Weight (kg)	54.6 (10.9)	54.4 (10.2)	54.8 (11.7)
BMI (kg/m^2^)	20.3 (3.4)	20.7 (3.4)	20.0 (3.3)
HSM1	2.3 (0.42)	2.3 (0.39)	2.2 (0.43)
HSM2	0.6 (0.76)	0.6 (0.75)	0.5 (0.77)
HSM5	−1.2 (0.78)	−1.2 (0.77)	−1.1 (0.79)


“Age 18”	Both	Females	Males
	N = 3175	N = 1755	N = 1420
	Mean (SD)	Mean (SD)	Mean (SD)
Age (years)	17.8 (0.38)	17.8 (0.38)	17.8 (0.39)
Height (cm)	171.5 (9.2)	165.4 (6.2)	178.9 (6.6)
Weight (kg)	67.1 (13.4)	62.6 (11.6)	72.7 (13.4)
BMI (kg/m^2^)	22.8 (4.0)	22.9 (4.0)	22.7 (3.9)
HSM1	2.4 (0.41)	2.4 (0.40)	2.4 (0.44)
HSM2	0.2 (0.84)	0.5 (0.82)	0.0 (0.80)
HSM5	−1.5 (0.85)	−1.3 (0.86)	−1.7 (0.78)

Abbreviations: SD (standard deviation), BMI (body mass index), HSM (hip shape mode).

### 2.4 The association of adult hip shape variants with adolescent hip shape

Genome-wide meta-analysis of adult hip shape (based on five cohorts: ALSPAC mothers, Framingham Osteoporosis Study, Osteoporotic Fractures in Men, Study of Osteoporotic Fractures and TwinsUK) identified nine SNPs across the 10 HSMs tested: five were associated with HSM1, three with HSM2 and one with HSM5 [10]. As previously described [10,19], genotypes were imputed to Haplotype Reference Consortium panel version 1 (ALSPAC, FOS, TwinsUK) or 1000 Genomes Project phase 1 version 3 (MrOS, SOF). Of participating adult cohorts Twins, SOF and MrOS DXA scans were acquired with Hologic scanners whereas FOS and ALSPAC (mothers and offspring) were acquired using Lunar scanner. These differences were reflected in skewed HSM1 scores, thus all genetic estimates and SDs were rescaled before the meta-analysis was conducted to standardize the results across the modes, as previously described [10].

Associations between adult SNPs and corresponding HSMs in adolescents were investigated separately at age 14 and 18 years. Linear regression analyses were performed using SNPTEST v2.5 assuming an additive genetic model, adjusting for age (in years) and sex. Additional analyses (adjusted for height and BMI) were also carried out. To adjust the level of statistical evidence, we applied a Bonferroni adjusted *p* value of 0.006 (α 0.05 divided by 8 SNPs tested), to account for the number of tests carried out. Given the units of the HSM variables, regression coefficients are expressed as change in the adult-standardised deviations from the mean hip shape per allele. Such effects are not that meaningful in themselves but do allow for comparison of effect estimates between the adult and adolescent analyses. In order to address the potential non-independence from the adult study (due the relationship between ALSPAC mothers and offspring), adult meta-analysis effects were re-calculated excluding ALSPAC mothers.

## 3 Results

**Fig. 1:**
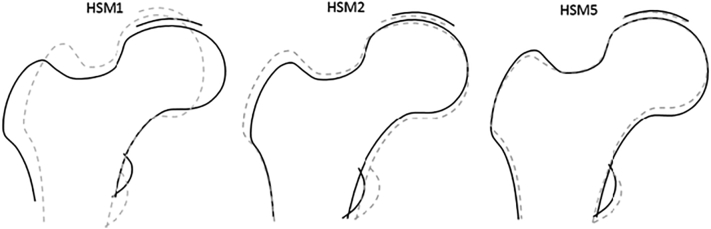
Variation described by hip shape modes 1, 2 and 5. Solid line represents 2 SDs above the mean HSM score, dashed line represents 2 SDs below the mean HSM score.

Our previous study in adults identified genome wide significant associations with HSM1, HSM2 and HSM5 [10]. Together, these three modes explain 60% of the total variance in hip shape. HSM1 indicates variation in aspect ratio of the upper femur; HSM2 reflects variation in FNW and size of femoral head and lesser and greater trochanters; HSM5 reflects variation in FNW, size of lesser trochanter and size of the inferior aspect of the femoral head proximal to lesser trochanter (Fig. 1).

Table 1 shows descriptive statistics of the study participants. At age 14 assessment clinic (mean age at attendance was 13.8 years), mean BMI was 20.3 kg/m^2^. At age 18 assessment clinic (mean age at attendance was 17.8 years), BMI was 22.8 kg/m^2^. As expected, males were taller than females at both time points. The positive mean scores for HSM1 and HSM2, and negative score for HSM5, reflect shape differences in adolescents relative to our reference SSM derived from adults (these differences reflect both variation in hip shape according to skeletal maturity, and differences in type of DXA scanner used in distinct cohorts e.g. pixel spacing with the Lunar Prodigy scanner used in ALSPAC leads to a distinct height to width ratio, the main shape feature of HSM1).

### 3.1 Association of adult hip shape variants with adolescent hip shape

Complete outcome and genetic data were available from 3550 and 3175 individuals at a mean age of 13.8 and 17.8 years, respectively. A total of 2618 individuals had both, genetic data and hip DXA images available at both time points. As expected, following exclusion of ALSPAC mothers from the adult meta-analysis we saw similar results in terms of magnitude of the effect compared with the originally reported beta coefficients, with slightly weaker strength of evidence observed in the analysis which excluded ALSPAC mothers (as reflected by larger *p* values) compared with the original study [10]. Throughout this manuscript, we will refer to GWAS results which excluded ALSPAC mothers. Table 2 shows the associations of adult hip shape SNPs with adolescent HSMs at ages 14 and 18. There was some weak evidence (*p* < 0.1) for effects in the same direction (as reported in adults) at either age 14 or 18 for all SNP-HSM associations tested. However, the majority had weaker effects in the current study of adolescents compared to the adult GWAS. Six of the nine associations met the Bonferroni-corrected *p*-value threshold (*p* < 0.006), these are described in more detail in the following sections.

**Table 2:** Hip shape meta-analysis results (adjusted for age and gender) and look-up in ALSPAC adolescent GWAS (adjusted for age and gender).

HSM	SNP	Locus/nearest gene	EA	EAF	Adult meta-analysis N = 12,823		Age 14 GWAS N = 3550		Age 18 GWAS N = 3175	
					Beta^a^	*p*	Beta^b^	*p*	Beta^c^	*p*
1	rs2158915	17q24.3/*SOX9*	G	0.35	−0.14	3.3 × 10^−23^	−0.054	**9.9 × 10**^**−8**^	−0.051	**3.3 × 10**^**−6**^
1	rs1243579	14q32.13/*GSC*	G	0.15	0.13	1.1 × 10^−13^	0.034	0.01	0.024	0.086
1	rs10743612	12p11.22/*KLHL42-PTHLH*	A	0.24	0.08	2.4 × 10^−8^	0.05	**1.1 × 10**^**−5**^	0.036	**0.003**
1	rs73197346	21q22.12/*RUNX1-MIR802*	C	0.14	−0.10	1.5 × 10^–7^^d^	−0.054	**2.4 × 10**^**−4**^	−0.035	0.026
1	rs59341143	4p15.33/*NKX3-2*	C	0.15	0.08	2.3 × 10^–6^^d^	0.023	0.102	0.025	0.093
2	rs1966265	5q35.2/*FGFR4*	T	0.38	0.13	2.3 × 10^−16^	0.051	0.018	0.072	**0.003**
2	rs6537291	4q31.21/*HHIP*	A	0.38	−0.079	4.2 × 10^−9^	−0.063	**0.001**	−0.069	**0.001**
2	rs1885245	9q33.1/*ASTN2*	G	0.40	0.075	4.4 × 10^−8^	0.044	0.019	0.013	0.545
5	rs2158915	17q24.3/*SOX9*	G	0.35	−0.093	8.7 × 10^−12^	−0.072	**1.6 × 10**^**−4**^	−0.10	**2.7 × 10**^**−6**^

Abbreviations: HSM (hip shape mode), SNP (single nucleotide polymorphism), EA (effect allele), EAF (effect allele frequency), *p* (*p* value). *p*-Values in bold meet a Bonferroni corrected α threshold = 0.006 (0.05/8).

a Results from hip shape meta-analysis in adults (excluding ALSPAC mothers), adjusted for age and gender.

b Results from hip shape GWAS in ALSPAC adolescents (age 14), adjusted for age and gender.

c Results from hip shape GWAS in ALSPAC adolescents (age 18), adjusted for age and gender.

d Please note that these SNPs reached genome-wide significance in the original adult study (with ALSPAC mothers included).

### 3.2 HSM1 and HSM5

**Fig. 2:**
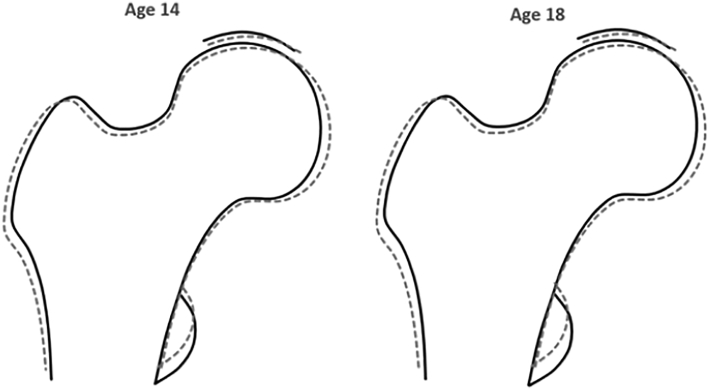
Composite effect of rs2158915 (located near *SOX9*) on HSM1 and HSM5. The overall effect was modelled for the minor allele (G), by entering the beta value for associations between rs2158915 with HSM1 and HSM5 at both adolescent time points, into SHAPE (see Table 2; beta estimates multiplied by 20 for illustrative purposes). Solid line represents baseline hip shape, dashed line represents effect allele result.

Five loci were associated with HSM1 in the previous adult GWAS (Table 2). One locus was associated with HSM5. This locus overlapped with HSM1 locus and so we report the same signal associated with both HSMs. Amongst the adult-associated variants, the strongest evidence for association in adolescents was seen for an intergenic SNP near *SOX9* at the 17q24.3 locus, rs2158915 (associated with HSM1 and HSM5 in adults). This variant was associated with HSM1 (age 14 β −0.054, *p* = 9.9 × 10^−8^; age 18 β −0.051, *p* = 3.3 × 10^−6^) and HSM5 (age 14 β −0.072, *p* = 1.6 × 10^−4^; age 18 β −0.10, *p* = 2.7 × 10^−6^), respectively, at both adolescent time points (consistent with the direction of the effect in adults and comparable effect sizes in case of association with HSM5; β −0.093, *p* = 8.6 × 10^−12^). On modelling the combined effect of rs2158915 on HSM1 and HSM5, findings were consistent across both time points (Fig. 2). The impact of the effect allele was reflected by smaller femoral head in the medial aspect, narrower femoral shaft, and larger lesser trochanter.

There was also strong evidence for an association between rs10743612 (an intergenic SNP between *KLHL42* and *PTHLH*; associated with HSM1 in adults; β 0.08, *p* = 2.4 × 10^−8^) and HSM1 at age 14 (β 0.05, *p* = 1.1 × 10^−5^), and at age 18 (β 0.036, *p* = 0.003). Compared with adults, the effect sizes were weaker in adolescents. Furthermore, rs73197346 (an intergenic SNP between *RUNX1* and *MIR802*; associated with HSM1 in adults; β −0.10 *p* = 1.5 × 10^−7^) was strongly related to HSM1 at age 14 (β −0.054, *p* = 2.4 × 10^−4^) with much weaker evidence observed at age 18 (β −0.035, *p* = 0.026). Compared with adults, the magnitude of effect at both adolescent time points was much weaker, with ≥ 50% reduction in beta coefficients.

### 3.3 HSM2

Three SNPs were associated with HSM2 in adults. There was strong evidence for an association between rs1966265 (a missense SNP of *FGFR4*) and HSM2 at age 18 (β 0.072, *p* = 0.003), with weaker evidence of an association observed at age 14 (β 0.051, *p* = 0.018). Compared with adult results (β 0.13, *p* = 2.3 × 10^−16^) the magnitude of effect was weaker at both time points in adolescents. We found rs6537291, an intergenic SNP upstream of *HHIP*, to also be associated with adolescent HSM2 at both age 14 and 18 years (β −0.063, *p* = 0.001; β −0.069, *p* = 0.001, respectively) and the magnitude of the effect was slightly weaker than the effect reported in adults (β −0.079, *p* = 4.2 × 10^−9^).

### 3.4 Sensitivity analyses

Given the associations between adult hip shape loci and other traits (including height and waist circumference) [10] we performed additional analyses adjusting for height and BMI. Compared with age and sex adjusted results (model 1) the results were essentially unchanged following additional adjustment for height (model 2) at both time points (Table 3). After additional adjustment for BMI (model 3), the majority of results remained unchanged, except for a small degree of attenuation in the association between HSM2 and rs1966265, a missense SNP of *FGFR4*. Compared with age and sex adjusted results, there was a slight reduction in the magnitude of effect at both time points (age 14 model 1: β 0.051, *p* = 0.018; model 3: β 0.043, *p* = 0.042; age 18 model 1: β 0.072, *p* = 0.003; model 3: β 0.063, *p* = 0.008).

**Table 3:** Associations between the selected SNPs and HSMs in adolescents (1) adjusted for age and gender (2) adjusted for age, gender and height and (3) adjusted for age, gender, and BMI.

HSM	SNP	EA	Age 14 (N = 3550)					
			Model 1		Model 2		Model 3	
			Beta	*p*	Beta	*p*	Beta	*p*
1	rs2158915	G	−0.054	9.9 × 10^−8^	−0.053	1.3 × 10^−7^	−0.053	1.6 × 10^−7^
1	rs1243579	G	0.034	0.010	0.033	0.013	0.035	0.009
1	rs10743612	A	0.050	1.1 × 10^−5^	0.051	8.3 × 10^−6^	0.050	1.2 × 10^−5^
1	rs73197346	C	−0.054	2.4 × 10^−4^	−0.051	4.3 × 10^−4^	−0.054	2.4 × 10^−4^
1	rs59341143	C	0.023	0.102	0.021	0.121	0.022	0.111
2	rs1966265	T	0.051	0.018	0.056	0.008	0.043	0.042
2	rs6537291	A	−0.063	0.001	−0.060	0.001	−0.063	4.2 × 10^−4^
2	rs1885245	G	0.044	0.019	0.038	0.035	0.047	0.009
5	rs2158915	G	−0.072	1.6 × 10^−4^	−0.075	7 × 10^−5^	−0.073	1.5 × 10^−4^


HSM	SNP	EA	Age 18 (N = 3175)					
			Model 1		Model 2		Model 3	
			Beta	*p*	Beta	*p*	Beta	*p*
1	rs2158915	G	−0.051	3.6 × 10^−6^	−0.051	3.2 × 10^−6^	−0.050	4.1 × 10^−6^
1	rs1243579	G	0.024	0.086	0.024	0.088	0.024	0.085
1	rs10743612	A	0.036	0.003	0.037	0.003	0.037	0.003
1	rs73197346	C	−0.035	0.026	−0.034	0.027	−0.034	0.027
1	rs59341143	C	0.025	0.093	0.025	0.097	0.025	0.088
2	rs1966265	T	0.072	0.003	0.074	0.002	0.063	0.008
2	rs6537291	A	−0.069	0.001	−0.069	9.5 × 10^−4^	−0.069	6.9 × 10^−4^
2	rs1885245	G	0.013	0.545	0.013	0.536	0.018	0.390
5	rs2158915	G	−0.10	2.7 × 10^−6^	−0.10	3.1 × 10^−6^	−0.10	1.9 × 10^−6^

Abbreviations: HSM (hip shape mode), SNP (single nucleotide polymorphism), EA (effect allele), *p* (*p* value).

## 4 Discussion

The identification of genetic variants for hip shape is important for increased understanding of the aetiology of OA and/or hip fracture. A GWAS has previously identified genetic variants associated with hip shape in adults using SSM (a method to describe overall hip shape). In this study we have investigated whether any of these genetic associations with hip shape are also present at younger ages. We found several consistent associations between previously reported adult hip shape variants and associations with the same hip shape variables in adolescents. There was strong evidence to suggest that these associations are present in as young as 14-year olds, suggesting genetic influences on hip shape are established by adolescence.

Genes implicated by the strongest associations with hip shape in adolescents, namely *SOX9* (associated with HSM1 and HSM5), *PTHLH* (associated with HSM1), *FGFR4* (associated with HSM2) and *HHIP* (associated with HSM2) are known to be involved in endochondral bone formation. However, whereas the latter is responsible for limb lengthening, the associations we observed were unaffected by height adjustment, suggesting an independent relationship with other aspects of hip shape and hip geometry. In attempting to define these, though identified using a hypothesis free approach, several HSMs represent recognisable geometric characteristics. For example, HSM1 indicates narrower aspect ratio of the upper femur, whereas both HSM2 and HSM5 reflect variation in the femoral head size and FNW. That genetic influences on HSM1 comprise effects on FNW is supported by findings from a previous GWAS meta-analysis, performed on an overlapping sample to our adult hip shape GWAS, in which rs6556301 (in modest LD with our rs1966265, R^2^ = 0.46) was also found to be related to FNW [20]. Similarly, *SOX-9* and *HHIP* loci were found to be associated with DXA-measured bone area [21].The genetic associations with hip shape in adolescents reported here may be relevant for future risk of age-related musculoskeletal disorders. Both FNW and variation in femoral head size have previously been found to be related to the risk of OA in later life. For example, wider FNW [22] and femoral head deformities [[23], [24], [25], [26]] were found to be associated with increased risk of hip OA in previous studies. As previously reported, a proxy SNP for intergenic SNP - rs10743612 – located between *KLHL42* and *PTHLH* (rs258394, R^2^ = 0.87) co-localized [10] with a signal previously reported to be associated with a greater risk of hip OA in the arcOGEN (OR = 1.14, *p* = 9.6 × 10^−5^) [27], whereas rs73197346 (an intergenic SNP between *RUNX1* and *MIR802*) was found to be associated with a reduced risk of hip OA in the UK Biobank (OR = 0.87, *p* = 0.006) [10]. Taken together, these findings support the suggestion that subtle morphological changes in hip shape, established in adolescence, predispose to the onset of hip OA [1]. Interestingly, in the most recent OA GWAS, performed in the full UK Biobank release, rs8067763 (located near *SOX9*, in modest LD with rs2158915 (R^2^ = 0.22), found to be associated with HSM1 and HSM5 in this study) has been found to be associated with knee OA [28] suggesting that genetic influences on endochondral bone formation may affect shape of other joints such as the knee.

Previous studies also suggest that hip shape, as measured by SSM, contributes to the risk of hip fracture. For example, Gregory et al. reported that the outline of the proximal femur, quantified on standard radiographs, was better at discriminating between hip fracture cases and controls compared with most bone mineral density (BMD) and all geometric measurements tested [2]. Similarly, Baker-LePain found that combining hip shape with BMD considerably improved the discrimination of incident hip fracture as compared with BMD alone [6]. A proxy SNP for rs2158915 (rs8082221, R^2^ = 1), associated with HSM1 and HSM5 in the present study, was also found to be associated with increased risk of hip fracture in adults (OR 1.26, *p* = 0.003, unpublished data) [10]. Although the same variant is also known to be associated with FN BMD [29], the present findings suggest that alterations in hip shape contribute to the relationship between this SNP and risk of hip fracture.

### 4.1 Strengths and limitations

This is the first study investigating the effect of adult hip shape genetic variants on adolescent hip shape. Studying younger individuals in a pre-disease state provides an important insight into the stages of development at which these established genetic variants likely exert their effects. Moreover, this approach ensures that any shape changes identified represent potential predisposing factors for hip OA, as opposed to changes occurring secondarily to OA. Another strength is that we used the same SSM as that in the genome-wide adult meta-analysis of hip shape [10], which enabled us to directly compare the effect estimates between two adolescent time points with those observed in adults. However, applying an SSM generated in adults to adolescent hips may have failed to capture the full extent of adolescent hip variation (for example when considering changes in shape before the fusion of growth plates).

Our approach was also potentially limited by the use of 2D images to study complex, multidimensional shape which could potentially bias the results due to hip rotation. It should be noted that rotation, if present, could arise due to both, anatomical variation or reflect associations with a disease (i.e. the degree of hip rotation required for DXA positioning may be limited in individuals with hip pain). However, any subtle positioning errors should be accounted for during Procrustes analysis. In addition, the deviation away from the mean in adolescent HSM scores was particularly noted for HSM1, which is likely to reflect scanner differences between ALSPAC and other cohorts in the adult reference set. Different pixel spacing in the Lunar Prodigy scanner relative to other scanners alters the aspect ratio (ratio between image height and width), and therefore HSM1 might reflect these differences. However, each GWAS was performed at an individual cohort level which is unlikely to have substantially affected our results.

We found that most of the variants seen in adults show some evidence of association in adolescents. Though a few of these did not reach a Bonferroni corrected threshold, the distribution of *p*-values suggests that most are likely to be associated, but smaller sample size (compared to the adult study) and potentially smaller effects in adolescents (or inflated effects in the original adult GWAS due to Winner's Curse) limited the power to find sufficient evidence for such associations. Hence further study of these SNPs in a larger sample of adolescents is recommended. Finally, whilst some of the variants identified in this study point towards nearby biologically relevant genes and might thus shed a light on biological pathways relevant to hip shape development and subsequent OA and fracture risk, further functional studies are necessary to confirm the causal variants and affected genes.

## 5 Conclusions

We explored the influence of established DXA-derived adult hip shape genetic variants on hip shape in ALSPAC adolescents at ages of 14 and 18 years. The majority of adult-identified associations show some evidence of consistent (albeit smaller) effects as early as age 14. Variants implicated in endochondral bone formation appear to influence hip shape in adolescents and adults, including those related to risk of hip OA and/or fracture in later life. Further analytical and molecular approaches are justified to bridge the knowledge gap and better understand mechanisms underlying these associations.

The following is the supplementary data related to this article.

Supplementary Table 1 Cross-correlations between the top ten HSMs at age 14 and 18 years in ALSPAC offspring, with outcome data available at both time points (N = 3188). Supplementary Table 1

## CRediT authorship contribution statement

Study design: LP and JT. Study conduct: LP, JT, DB and MF. Data collection: JG, RA, MF. Data analysis: MF. Data interpretation: LP, JT, JG, RA, DB and MF. Drafting manuscript: MF and LP. Revising manuscript content: LP, JT, DB, JG, RA, NEL, CO, UPK, DK and MF. Approving final version of manuscript: LP, JT, DB, JG, RA, NEL, CO, UPK, DK and MF. MF takes responsibility for the integrity of the data analysis.

## Declaration of competing interest

All authors state that they have no conflicts of interest.
