# Supplementary material for: The influence of adult hip shape genetic variants on adolescent hip shape: Findings from a population-based DXA study
Source: Bone. 2021 Feb;143:115792. doi: 10.1016/j.bone.2020.115792 (PMC7809624; doi:10.1016/j.bone.2020.115792)
Supplement: Supplementary Table 1 — Cross-correlations between the top ten HSMs at age 14 and 18 years in ALSPAC offspring, with outcome data available at both time points (N = 3188). [file mmc1.docx]

**The influence of adult hip shape genetic variants on adolescent hip shape: findings from a population-based DXA study**

Authors: Monika Frysz PhD^1,2^, Denis Baird PhD^2^, Jenny S Gregory PhD^3^, Richard M Aspden PhD^3^, Nancy E Lane MD^4^, Claes Ohlsson MD PhD^5,6^, [Ulrika Pettersson-Kymmer](https://www.ncbi.nlm.nih.gov/pubmed/?term=Pettersson-Kymmer%20U%5BAuthor%5D&cauthor=true&cauthor_uid=32067027) MD PhD^7^, David Karasik PhD^8,9^, Jonathan H Tobias MD PhD^1,2^, Lavinia Paternoster PhD^2^

Supplementary material

**Supplementary Table 1** Cross-correlations between the top ten HSMs at age 14 and 18 years in ALSPAC offspring, with outcome data available at both time points (N= 3,188)

|  | HSM1_age14 | HSM2_age14 | HSM3_age14 | HSM4_age14 | HSM5_age14 | HSM6_age14 | HSM7_age14 | HSM8_age14 | HSM9_age14 | HSM10_age14 |
| --- | --- | --- | --- | --- | --- | --- | --- | --- | --- | --- |
| HSM1_ age18 | 0.79 |  |  |  |  |  |  |  |  |  |
| HSM2_ age18 | 0.24 | 0.75 |  |  |  |  |  |  |  |  |
| HSM3_ age18 | 0.26 | 0.38 | 0.40 |  |  |  |  |  |  |  |
| HSM4_ age18 | 0.07 | 0.17 | 0.25 | 0.55 |  |  |  |  |  |  |
| HSM5_ age18 | 0.50 | 0.29 | 0.10 | 0.11 | 0.62 |  |  |  |  |  |
| HSM6_ age18 | -0.22 | -0.09 | -0.05 | -0.15 | -0.21 | 0.47 |  |  |  |  |
| HSM7_ age18 | 0.10 | 0.12 | 0.23 | 0.12 | 0.01 | -0.08 | 0.51 |  |  |  |
| HSM8_ age18 | -0.15 | -0.05 | 0.08 | 0.06 | -0.03 | 0.08 | -0.22 | 0.54 |  |  |
| HSM9_ age18 | -0.05 | 0.18 | 0.06 | 0.17 | 0.14 | -0.23 | 0.12 | -0.19 | 0.45 |  |
| HSM10_ age18 | -0.01 | 0.04 | 0.03 | -0.13 | -0.01 | 0.07 | 0.05 | 0.09 | -0.07 | 0.30 |
